# Hydrophilic-treated plastic plates for wide-range analysis of Giemsa-stained red blood cells and automated *Plasmodium* infection rate counting

**DOI:** 10.1186/s12936-017-1975-9

**Published:** 2017-08-08

**Authors:** Muneaki Hashimoto, Shouki Yatsushiro, Shohei Yamamura, Masato Tanaka, Hirokazu Sakamoto, Yusuke Ido, Kazuaki Kajimoto, Mika Bando, Jun-ichi Kido, Masatoshi Kataoka

**Affiliations:** 1 Health Research Institute, National Institute of Advanced Industrial Science and Technology (AIST), 2217-14, Hayashi-cho, Takamatsu, Kagawa 761-0301 Japan; 2 Department of Periodontology and Endodontology, Institute of Health Biosciences, The University of Tokushima Graduate School, Institute of Health Biosciences, 3-18-15 Kuramoto, Tokushima, 770-8504 Japan; 3 Department of Biochemistry and Molecular Biology, Graduate School and Faculty of Medicine, The University of Tokyo, 7-3-1, Hongo, Bunkyo-Ku, Tokyo 113-0033 Japan

**Keywords:** Malaria, Diagnosis, Automation, Hydrophilic treatment, Giemsa-staining

## Abstract

**Background:**

Malaria is a red blood cell (RBC) infection caused by *Plasmodium* parasites. To determine RBC infection rate, which is essential for malaria study and diagnosis, microscopic evaluation of Giemsa-stained thin blood smears on glass slides (‘Giemsa microscopy’) has been performed as the accepted gold standard for over 100 years. However, only a small area of the blood smear provides a monolayer of RBCs suitable for determination of infection rate, which is one of the major reasons for the low parasite detection rate by Giemsa microscopy. In addition, because Giemsa microscopy is exacting and time-consuming, automated counting of infection rates is highly desirable.

**Results:**

A method that allows for microscopic examination of Giemsa-stained cells spread in a monolayer on almost the whole surface of hydrophilic-treated cyclic olefin copolymer (COC) plates was established. Because wide-range Giemsa microscopy can be performed on a hydrophilic-treated plate, the method may enable more reliable diagnosis of malaria in patients with low parasitaemia burden. Furthermore, the number of RBCs and parasites stained with a fluorescent nuclear staining dye could be counted automatically with a software tool, without Giemsa staining. As a result, researchers studying malaria may calculate the infection rate easily, rapidly, and accurately even in low parasitaemia.

**Conclusion:**

Because the running cost of these methods is very low and they do not involve complicated techniques, the use of hydrophilic COC plates may contribute to improved and more accurate diagnosis and research of malaria.

**Electronic supplementary material:**

The online version of this article (doi:10.1186/s12936-017-1975-9) contains supplementary material, which is available to authorized users.

## Background

Parasitaemia, the count of *Plasmodium* spp. cells in red blood cells (RBCs), is a commonly used parameter in malaria diagnosis. Since 1904, microscopic examination of thick and thin Giemsa-stained blood films (hereafter, ‘Giemsa microscopy’) has been the gold standard for malaria diagnosis [1]. Giemsa microscopy is regarded as the most suitable diagnostic technique for malaria because it is inexpensive, quantitative, and can differentiate between various infective malaria species [2]. Because thick blood films contain several layers of RBCs, many parasites can be analysed, thus making it useful for diagnosing patients with low parasitaemia, particularly compared with thin blood smear analysis. However, accurate counting of parasitaemia in thick film analysis is difficult even for skilled microscopists [3], and diagnosis using thick films is not suitable for differentiating between parasitic species. Considering these limitations, analysis of thin blood smears may be important for accurate malaria diagnosis.

A rapid diagnostic test (RDT) or polymerase chain reaction (PCR)-based diagnosis are conventionally performed for malaria. RDT immunologically detects the malarial antigen in a simple manner with a detection limit similar to that of Giemsa microscopy [4–6]. However, RDT is relatively costly and shows high rates of false-positive and (or) false-negative results. PCR-based diagnosis shows higher specificity and sensitivity in identifying and differentiating malaria at the species level. However, because the diagnostic costs are high, this method is often not practical for use in endemic areas [5, 7, 8]. Furthermore, neither RDT nor PCR can be used for quantitative analysis of parasitaemia levels [5, 9].

The procedure for Giemsa microscopy consists of the following steps: preparation of a thin blood smear, fixation and staining of the smear, and microscopic observation of the parasites in RBCs. For quantification of parasitaemia, a monolayer region of the blood smear, where RBCs are sparsely distributed, is examined. This ensures identification and accurate counting of the parasites. However, although skilled technicians prepare the blood smears, only a small proportion of the smear will become a monolayer, thereby severely limiting the area (and number of RBCs) available for analysis. The low sensitivity of this method is its main disadvantage [10]. A method that allows for microscopic examination of Giemsa-stained cells spread in a monolayer on almost the whole surface of hydrophilic-treated cyclic olefin copolymer (COC) plates was established. Thin blood smears provide much clearer images than thick blood films and can be used to identify parasitic species; however, thin smears show lower sensibility. The method developed in this study may compensate for the disadvantages of thin smears and improve the accuracy of malaria diagnosis. Furthermore, a method for accurate automated quantification of RBCs and infecting *Plasmodium* parasites, stained with a fluorescent nuclear staining dye, on hydrophilic-treated plates by employing an image software analysis tool instead of Giemsa staining and microscopic examination was reported.

## Methods

### Malaria culture and detection of parasite-infected red blood cells

*Plasmodium falciparum* strain 3D7 was cultured as previously described [11]. Blood from a healthy donor was collected in BD Microtainer Tubes containing K2E (K_2_EDTA) (BD Biosciences, Franklin Lakes, NJ, USA). Giemsa microscopy was also performed as described earlier [11]. For automated counting of infected parasites, the parasite-infected RBCs were stained with SYTO21 (Thermo Fisher Scientific, Waltham, MA, USA) at a final concentration of 5 µM for 10 min. Bright field and fluorescence images of parasite-infected RBCs stained with SYTO21 were acquired using an inverted fluorescence microscope (DM1L, Leica Microsystems, Wetzlar, Germany) with a digital camera (MC120 HD, Leica Microsystems) and analysed using MetaMorph Offline software (ver. 7.8, Molecular Devices, Sunnyvale, CA, USA).

### Preparation of hydrophilic-treated COC plate surfaces

Plastic plates (25 × 75 mm) made of cyclic olefin copolymer (COC, Shin-Etsu Polymer Co. Ltd., Tokyo, Japan) were rendered hydrophilic by reactive ion-etching treatment using a SAMCO RIE system (SAMCO Inc., Tokyo, Japan) [11–13]. The treatment was performed for 15 min. The effect of reactive ion etching on the plate surface was examined by measuring the contact angle of water on the plate surface using a contact angle meter (Kyowa Interface Science Co. Ltd., Saitama, Japan). The contact angle of the plates was 18.2°, and the effect on the plates lasted for more than 2 weeks.

### Statistical analysis

Statistical analysis was performed with SigmaPlot ver. 12 (Systat Software, Inc., San Jose, CA, USA) using a Student’s *t* test.

## Results

### Preparation of a uniform monolayer of Giemsa-stained red blood cells on hydrophilic-treated COC plates

**Fig. 1:**
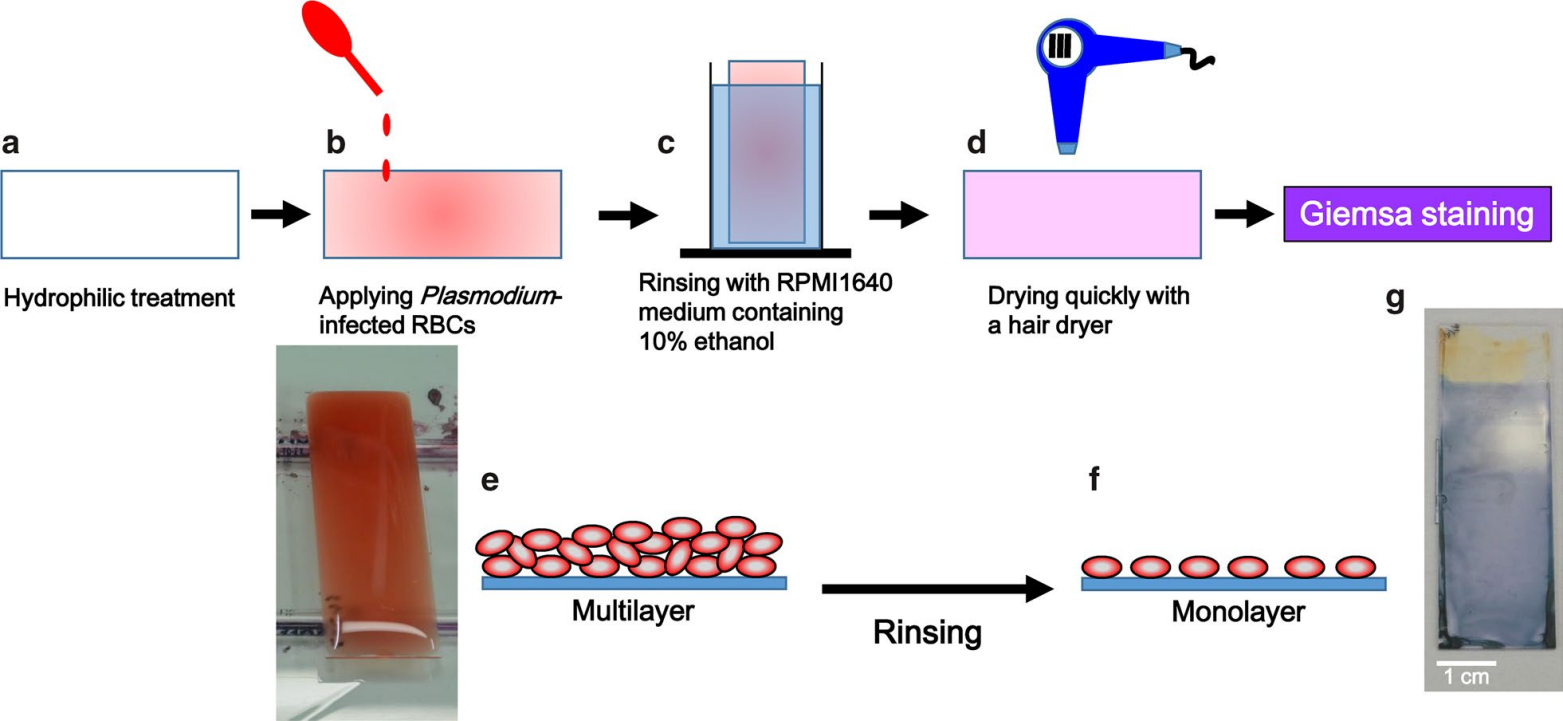
Protocol for preparing Giemsa-stained RBC monolayers on hydrophilic-treated COC plates. After hydrophilic treatment of COC plates (**a**), parasite-infected RBCs were added to the plates (**b**). A picture of a plate is also shown. Cells were allowed to bind to the plates. After removing non-adherent cells (**c**), the plates were dried rapidly (**d**). The cells were distributed as a monolayer on the plates. Schematic cross-sectional images of RBCs on a COC plate (**e**) before and (**f**) after rinsing. **g** A hydrophilic-treated COC plate after Giemsa staining

Because the adhesiveness of cells to plastic plates treated with reactive ions is increased, the hydrophilic-treated COC plates could be used for preparation of RBC monolayers. As described in Methods, reactive ion-etching treatment was performed on slide-glass shaped COC plates (Fig. 1a). 3 ml of *P. falciparum*-infected RBCs (1% parasitaemia) diluted to 1% of haematocrit with RPMI1640 medium were dropped onto the hydrophilic-treated COC plates. The plates were then left undisturbed for 10 min to allow the RBCs to settle and adhere onto the plate (Fig. 1b, e). Non-adherent RBCs were removed by rinsing the plate for 10 s with a medium containing 10% ethanol (v/v), which also facilitated the drying of adherent RBCs in subsequent steps, and the RBCs then formed a monolayer on the plate surface (Fig. 1c, f). RBCs detached from the plate if higher concentrations of ethanol were used in the rinsing medium. The monolayer, which covered the entire plate, was immediately dried using a hair dryer (Fig. 1d) to avoid disrupting the morphology of the RBCs or the parasites. The dried plates were stained by the conventional Giemsa staining method. Figure 1g shows a hydrophilic-treated COC plate after Giemsa staining. The RBCs were stained uniformly across the plate, which was significantly different from that observed using the conventional technique, in which a thin blood smear is stained. Most of the RBCs were attached to the plates because of immediate drying using a hair dryer. However, if they were not dried immediately, the RBCs were removed by methanol treatment. A hair dryer with a powerful motor should be used to apply air from directly above the plate.

**Fig. 2:**
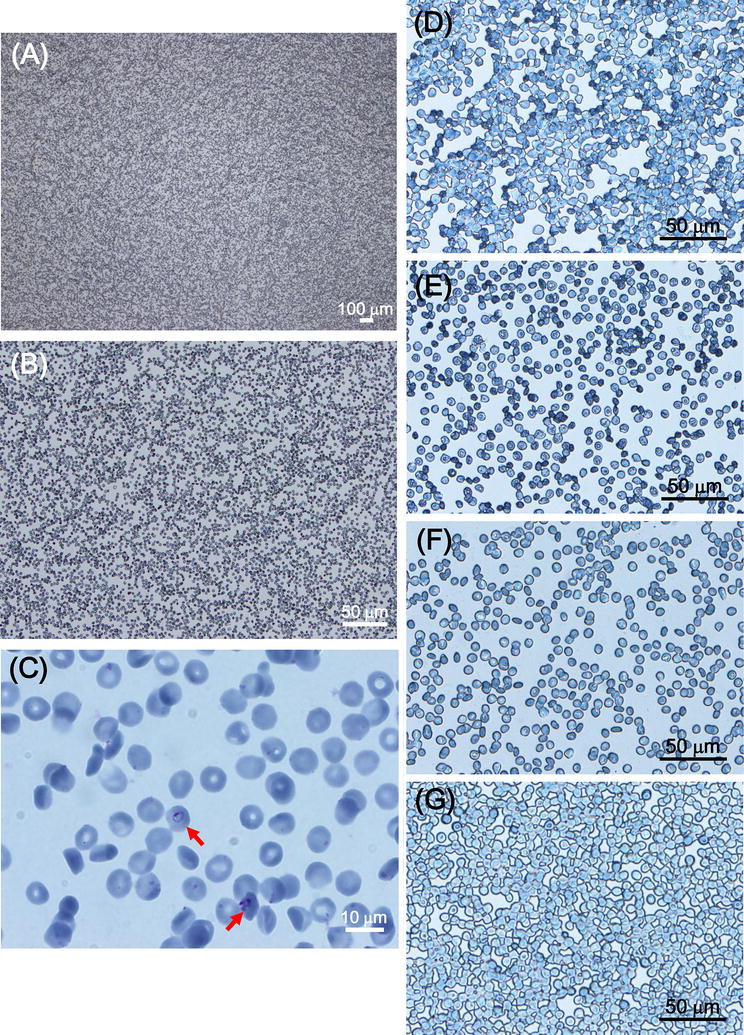
Representative images of Giemsa-stained hydrophilic-treated COC plates. **A**–**C** Representative microscopic images of Giemsa-stained parasite-infected RBCs on a hydrophilic-treated COC plate. Magnification **A** ×50, **B** ×200, **C** ×1000, oil immersion. *Arrows* indicate *Plasmodium*-infected cells. **D**–**F** Representative microscopic images of Giemsa-stained RBCs on hydrophilic-treated plates with water contact angles of **D** 38.6°, **E** 31.0°, and **F** 18.2°. **G** Representative microscopic image of Giemsa-stained RBCs on a glass slide. The sample was prepared using the protocol described in the legend to Fig. 5. The water contact angle of the glass slide was 7.8°

When the plates were observed microscopically, a monolayer of non-aggregated RBCs was visualized all over the plate surface (Fig. 2A, B). Parasite-infected RBCs were observed using an oil immersion lens (Fig. 2C). The contact angle of water on the hydrophilic-treated COC plates was also measured. The concept of the contact angle is described in the Additional file 1: Figure S1). Figure 2F shows a Giemsa-stained plate with a contact angle of 18.2° (Fig. 2A–C, F); in contrast, the contact angle of the untreated plate was 111.9°. The RBCs formed a monolayer and were sparsely distributed across the hydrophilic-treated plate, which enabled parasite identification and accurate enumeration. Next, plates with a contact angle of 38.6° (Fig. 2D) or 31.0° (Fig. 2E) using weaker reactive ion etching were prepared, and these plates were used for Giemsa microscopy. There was a dense population of RBCs on the plate, with a contact angle of 38.6°, which was not suitable for microscopic analysis (i.e., most RBCs were aggregated). However, cell distribution on the plate with a contact angle of 31.0° was well suited for Giemsa microscopy. Giemsa staining on the whole surface of a glass slide was also performed as described in Fig. 1b–d. The contact angle of water on the conventional glass slide was 7.8°. The RBCs were aggregated and did not form a monolayer suitable for Giemsa microscopy on the slide. Additionally, diluting the RBCs to 0.1% haematocrit did not reduce aggregation.

**Fig. 3:**
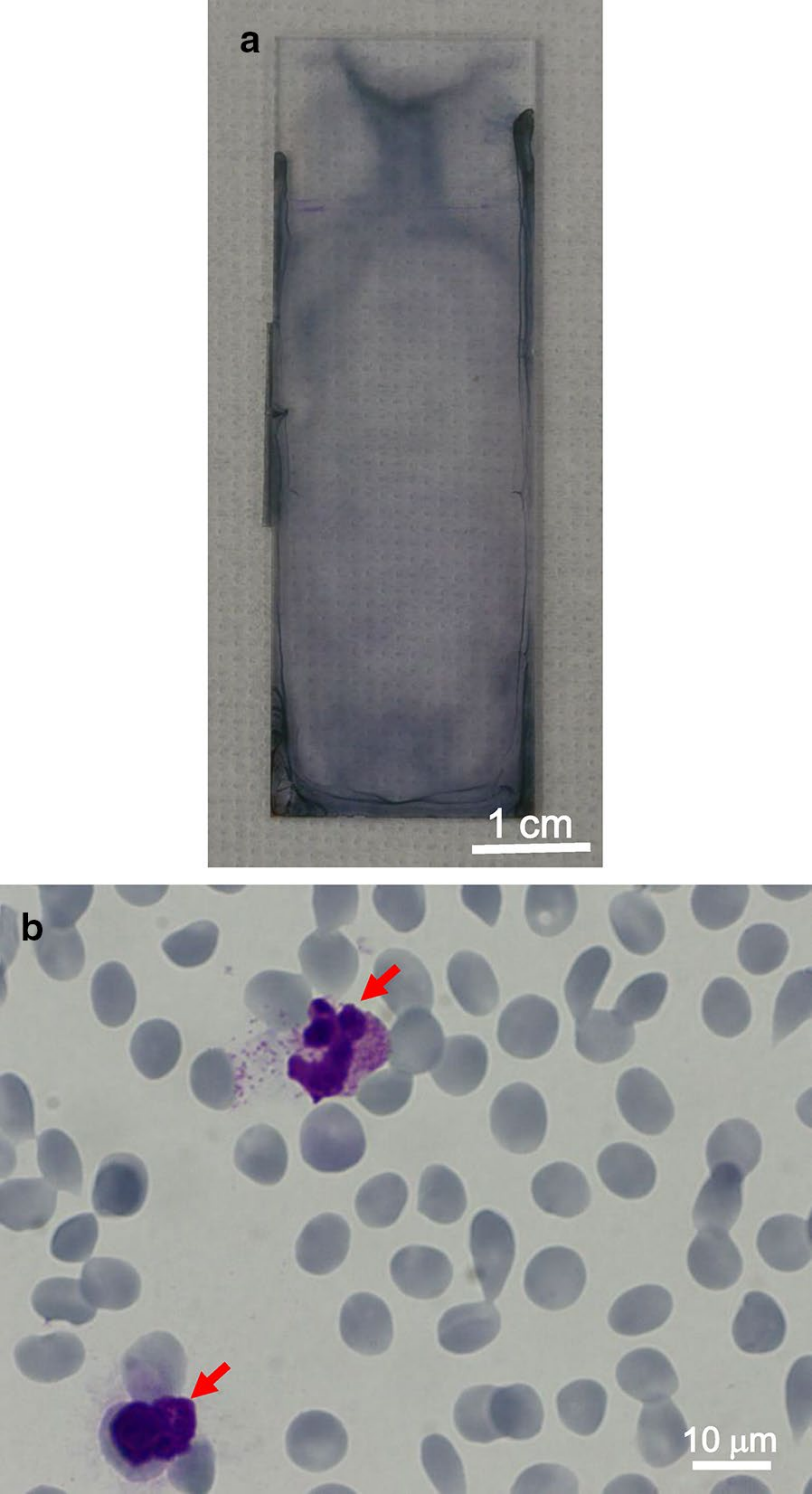
Representative images of Giemsa-stained hydrophilic-treated COC plates. **a**, **b** Blood from a healthy donor was applied to the hydrophilic-treated plate followed by Giemsa staining. Hydrophilic-treated COC plate after Giemsa staining (**a**). Representative microscopic images of Giemsa-stained blood cells on a hydrophilic-treated COC plate at a magnification of ×1000, oil immersion (**b**). *Arrows* indicate leukocytes

Next, whether a uniform monolayer formed on the hydrophilic-treated plate when whole blood was used as the sample was evaluated. In this study, blood from a healthy donor was diluted 100-fold with RPMI 1640 medium followed by staining with Giemsa solution, as described in Fig. 1. Figure 3a shows the hydrophilic-treated COC plate after Giemsa staining, which is similar to the plate applied for cultured *Plasmodium*-infected RBCs (see Fig. 1g). Microscopic analysis with 1000× oil immersion revealed that RBCs formed a uniform monolayer on the plate and Giemsa-stained leukocytes formed a monolayer (Fig. 3b). These results indicate that RBCs in whole blood form a uniform monolayer on the plate and can be used for Giemsa staining. Therefore, the method may be suitable for use with Giemsa microscopy to diagnose patients with malaria.

### Automated RBC counting on hydrophilic-treated COC plates

**Fig. 4:**
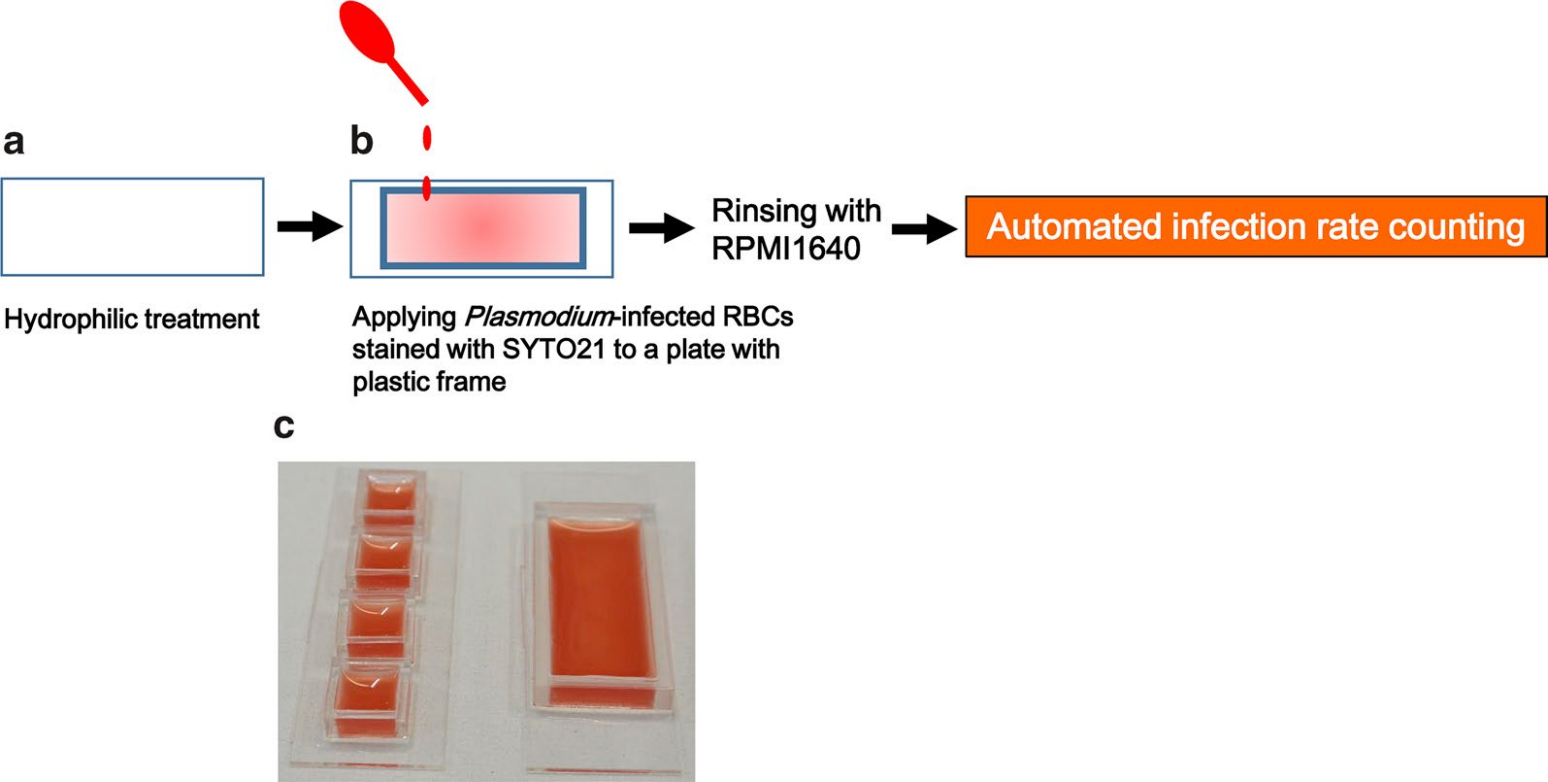
Protocol for automated counting of *Plasmodium* infection rate. After hydrophilic treatment of COC plates (**a**), parasite-infected RBCs stained with SYTO21 were added to the plate with various sizes of plastic frames (**b**). A picture of a plate is also shown (**c**). Cells were allowed to bind to the plate. After removing non-adherent cells, the cells were automatically counted using an inverted fluorescence microscope and MetaMorph Offline software

Determination of the parasite infection rates is essential for malaria diagnosis as well as for research, but counting the number of RBCs and infected parasites by microscopic examination is laborious and time-consuming. Hence, a method for automated infection rate counting on hydrophilic-treated COC plates was developed. RBCs diluted to 1% hematocrit with RPMI1640 medium were added to the hydrophilic-treated COC plate with frame(s) composed of plastic, allowing 10 min for the RBCs to settle and adhere to the COC plate (Fig. 4a, b). Plastic frames of various sizes can be attached with double-sided tape and nail polish, depending on the experimental purpose (Fig. 4c). Non-adherent RBCs were removed by rinsing with medium, and the remaining RBCs formed a monolayer on the plate surface.

**Fig. 5:**
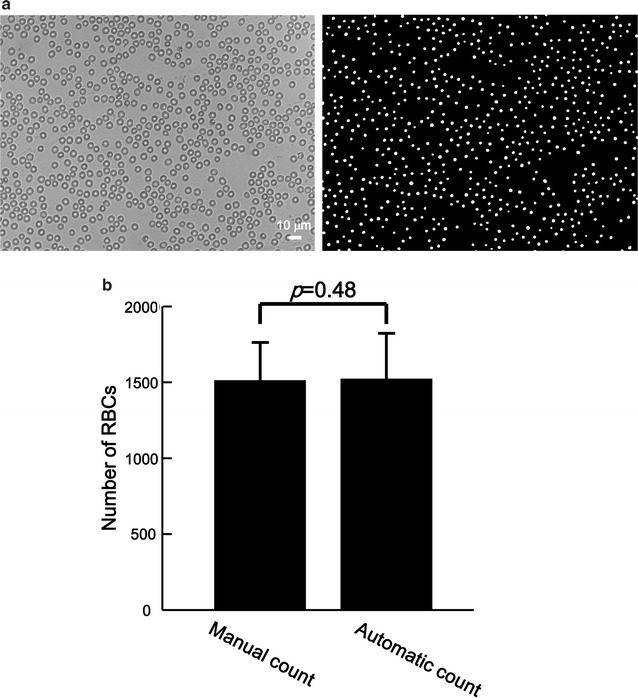
Automated counting of red blood cells. **a** Representative bright field image of RBCs on the hydrophilic-treated COC plate (*left panel*). RBCs are specifically detected and automatically counted with MetaMorph Offline software. The detected RBCs are presented as *white dots* (*right panel*). **b** The numbers of RBCs on the plates were counted manually or automatically. The resultant numbers were not significantly different between the two methods

Figure 5a (left panel) shows a bright field image of an RBC monolayer on the COC plate. RBCs were sufficiently dispersed for accurate counting. The dimensions of the RBCs were very similar (approximately 7 µm in diameter), and the colour of the centre of the RBCs appeared white in the picture taken with a digital microscopic camera. These features were useful for automated RBC counting with a software tool. RBCs were specifically detected by integrated morphometry analysis (total area > 25 and 0.8 < shape factor < 1.0) in MetaMorph Offline software (ver. 7.8, Molecular Devices, Sunnyvale, CA, USA) (Fig. 5a, right panel). Concerning the five bright field images, the number of RBCs was counted manually and automatically to compare the values obtained by the two methods (Fig. 5b). The number of RBCs was not significantly different between the two counting methods (*p* = 0.48). The fluorescence signal intensity of the reticulocytes stained with SYTO21 (a dye used for staining the parasites, see below) was much weaker and larger in size than that of the parasites. Because the signals corresponding to reticulocytes were omitted in the analysis using MetaMorph software, reticulocytes did not show false-positive results. Howell–Jolly bodies were not observed in this study. It required less than 1 min to perform the automated counting. Taken together, these data indicate that accurate automated RBC counting can be performed easily and quickly with the hydrophilic-treated COC plate surfaces and MetaMorph Offline software.

### Automated *Plasmodium*-infected red blood cell detection and counting on hydrophilic-treated COC plates

**Fig. 6:**
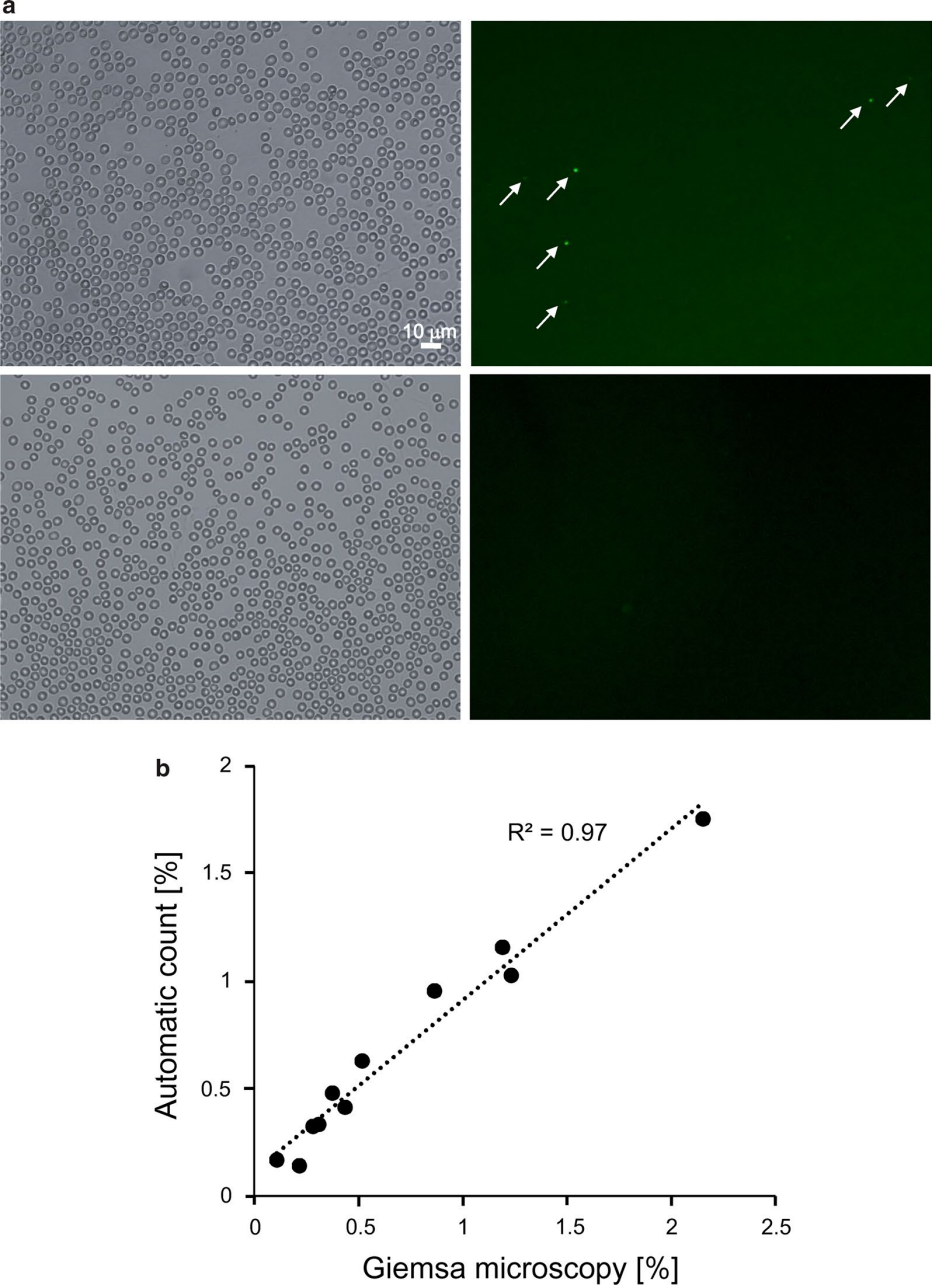
Automated counting of parasites. **a** Representative bright field image of RBCs on hydrophilic-treated plates (*left panels*). Fluorescence images of the same field (*right panels*). Images of *Plasmodium*-infected or uninfected RBCs stained with SYTO21 are shown in the *upper and lower panels*, respectively. Fluorescence signals from the parasite nuclei were detected (*arrows on upper left panel*). **b** The infection rate of parasite-infected cells with various cell infection frequencies was analysed by Giemsa microscopy or by the automated counting method to determine the correlation between the two methods

Next, the automated counting of *Plasmodium*-infected RBCs on hydrophilic-treated COC plates was attempted. Since, unlike parasites, RBCs do not have nuclei, it is possible to detect parasite-infected RBCs by nuclear staining. SYTO21, a cell-permeant green fluorescent dye, was used for staining of the parasite nucleic acids. The parasite-infected RBCs, suspended in the medium containing SYTO21, were added to hydrophilic-treated plates, followed by staining for 10 min and removal of non-adherent RBCs. Non-synchronized parasites, in which more than 60% of the parasites were in the ring stage and whose fluorescence signals were weaker than those of parasites in other stages, were used in this study. Figure 6a (upper left panel) shows a representative bright field image of the parasite-infected RBCs. The RBCs formed a monolayer, which was then available for automated counting. Figure 6a (upper right panel) presents a fluorescence image of the same field. Infected *Plasmodium* nuclei stained with SYTO21 were detected (arrows). The signals were not identified in uninfected RBCs (Fig. 6a, lower panels).

Automated counting of the detected fluorescence signals was possible using the MetaMorph Offline software, and the automated counting was performed with Integrate Morphometry Analysis (total area > 10, and 0.4 < shape factor < 1.0). When the data obtained were combined with the number of RBCs automatically counted as described in Fig. 5, the infection rate could be calculated easily $\left[ {{\text{infection rate }}\left( \% \right) = {\text{number of fluorescence signals}}/{\text{number of RBCs}} \times 100} \right]$. Eleven *Plasmodium*-infected RBC samples with various infection rates (from 0.10 to 2.15%) were analysed by Giemsa microscopy and the automated counting technique to compare the two counting methods (Fig. 4b). The infection rates calculated by these two methods exhibited a positive correlation (R^2^ = 0.97). These results indicate that automated counting of the parasite infection rate is possible without preparation of a thin blood smear, Giemsa staining, or microscopic observation.

## Discussion

Giemsa microscopy has been the gold standard for malaria diagnosis for more than 100 years. Since the area of the blood smear on the glass slide wherein the RBCs are in a monolayer is quite small, the sensitivity of *Plasmodium* detection is poor. In this study, the method for preparing RBC monolayers for Giemsa microscopy was improved. The number of RBCs available for analysis, which formed a monolayer and were sparsely distributed, was significantly higher on the hydrophilic-treated COC plates than on conventional glass slides. Interestingly, Le et al. [14] reported a method for automated counting of parasitaemia in Giemsa-stained thin blood smears. Therefore, the method might be useful for the accurate diagnosis of malaria in patients with low parasitaemia. However, because Giemsa microscopy must be conducted using a 100× oil immersion lens, development of a complex observation device for a microscope with an automatically mobile stage may be required for automated Giemsa microscopy analysis. One of the advantages of conducting Giemsa microscopy with thin blood smears is the ability to identify parasitic species. On thin smears, both parasites and infected RBCs (e.g. size, Schüffner’s dots) can be examined. However, it is difficult for even skilled microscopists to identify parasitic species by observation of only one or two parasites. Several infected RBCs should be examined before definite diagnosis. Because a large number of RBCs can be counted on a hydrophilic-treated COC plate, diagnostic accuracy may be improved.

Furthermore, a method for automated counting of parasite-infected cells without Giemsa staining using hydrophilic-treated COC plates are developed. Calculation of the infection rate is essential to cultivate or investigate the parasite, but this is laborious and time-consuming work. Further, operators have to practice the conventional method extensively for accurate infection rate calculation. The running cost of the automated counting method is very low (about 0.3 USD per COC plate). When large plastic frame(s) for applying RBCs are employed, huge numbers of RBCs on a plate can be analysed. In contrast, because some smaller frames can be placed on a plate, certain significant conditions (drug concentration or reaction time) can be analysed on the plate, which might be useful for screening of new drug candidates. Leucocytes containing nuclei could interfere with the determination of parasitaemia in patients with malaria. However, because the size of the leucocyte nuclei is greater than that of the parasite nuclei, these can be distinguished using the MetaMorph Offline software after detection of SYTO21 signals. Furthermore, over 99.9% of leucocytes can be removed with a push column in fieldwork situations [12]. Therefore, the automated counting method could be used in diagnostic settings.

Conducting automated infection rate counting with SYTO21 may not be practical. Fluorescence microscopy is costly, a freezing system is required to maintain SYTO21 stability, and a purchase route for reagents is required. Researchers in laboratories with access to this equipment can quickly calculate parasitic infection rates at a low cost. In contrast, Giemsa microscopy can be performed on hydrophilic-treated plates and requires a power supply for a hair dryer, making this method suitable for field settings. Because approximately 20 min are required to prepare a plate with a Giemsa-stained RBC monolayer, the method can be performed at the point-of-care. Previously, hydrophilic-treated plates were transported to an endemic country (Gulu, Uganda) in high temperature and humidity environments. The samples remained stable for more than 2 weeks. Further, because this method uses quick drying with a hair dryer, the risk of contamination with patient blood is reduced and diagnosis can be safely conducted.

## Conclusions

Taken together, hydrophilic-treated COC plate surfaces can be used not only for wide-range analysis by Giemsa microscopy, but also for automated and accurate infection rate counting without Giemsa staining.

## Additional file

**Additional file 1: Figure S1.** Concept of contact angle. Contact angle measurement is used to evaluate surface energy, wettability, and adhesion of low-surface energy materials (Subedi DP, The Himalayan Physics, 2011). Illustration of water on a hydrophobic surface (left) and hydrophilic surface (right) are shown. Water on the hydrophobic surface showed a larger contact angle (ɵ > 90°), whereas water on the hydrophilic surface show a smaller contact angle (ɵ < 90°). Contact angle is determined from the difference between cohesive and adhesive forces of solid and liquid molecules.

## Abbreviations

COC: cyclic olefin copolymer
RBC: red blood cell
