# Supplementary figures and images for: Hydrophilic-treated plastic plates for wide-range analysis of Giemsa-stained red blood cells and automated Plasmodium infection rate counting
Source: Malar J. 2017 Aug 8;16:321. doi: 10.1186/s12936-017-1975-9 (PMC5549322; doi:10.1186/s12936-017-1975-9)

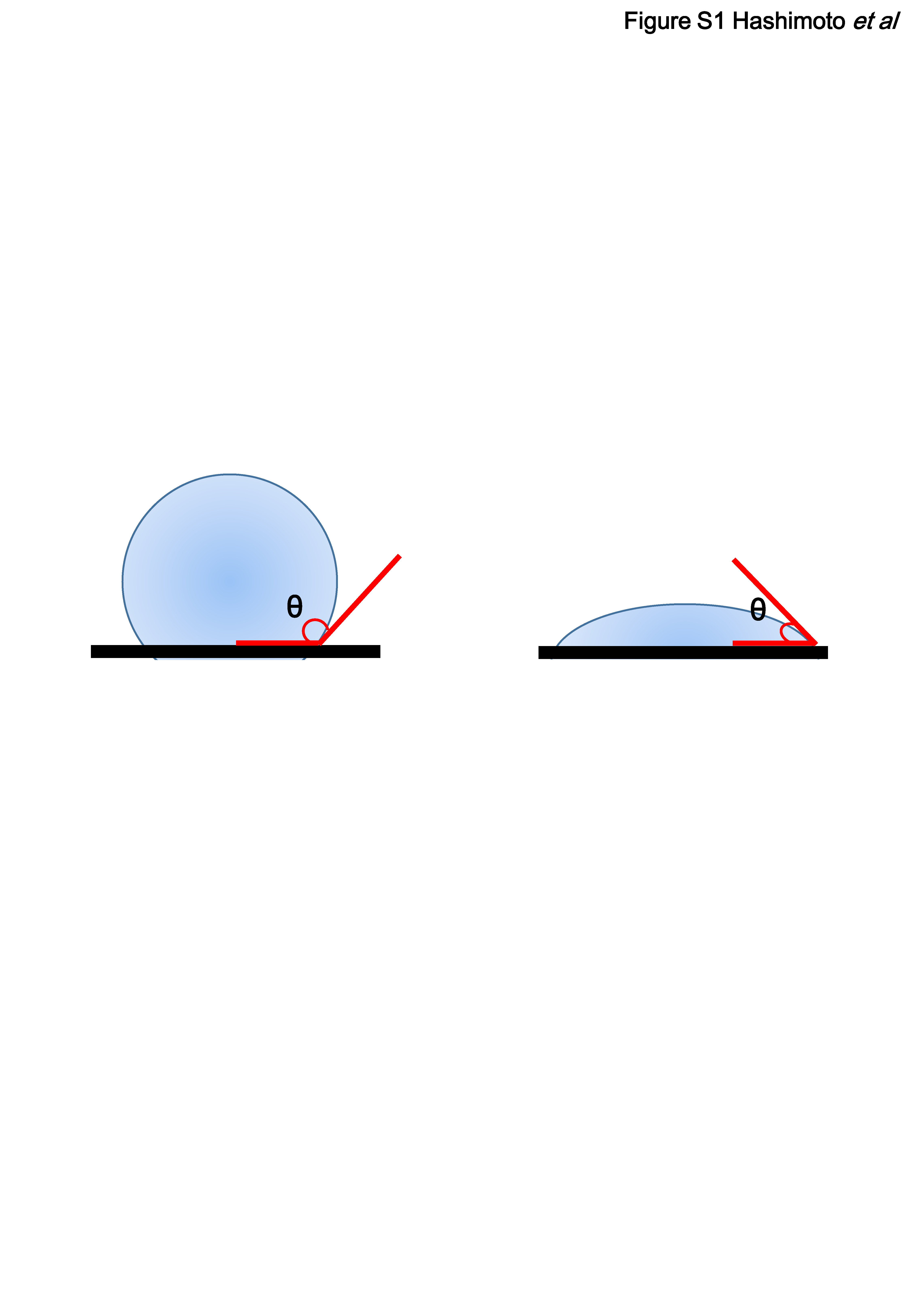

Supplement: Supplementary file 1 — Additional file 1: Figure S1. Concept of contact angle. Contact angle measurement is used to evaluate surface energy, wettability, and adhesion of low-surface energy materials (Subedi DP, The Himalayan Physics, 2011). Illustration of water on a hydrophobic surface (left) and hydrophilic surface (right) are shown. Water on the hydrophobic surface showed a larger contact angle (ɵ > 90°), whereas water on the hydrophilic surface show a smaller contact angle (ɵ < 90°). Contact angle is determined from the difference between cohesive and adhesive forces of solid and liquid molecules. [file 12936_2017_1975_MOESM1_ESM.tiff]
